# Cannabis Use Among Remote Indigenous Australians: Opportunities to Support Change Identified in Two Waves of Sampling

**DOI:** 10.3389/fpubh.2018.00310

**Published:** 2018-11-02

**Authors:** Veronica E. Graham, Alan R. Clough

**Affiliations:** ^1^College of Public Health, Medical and Veterinary Sciences, James Cook University, Cairns, QLD, Australia; ^2^Australian Institute of Tropical Health and Medicine, James Cook University, Cairns, QLD, Australia

**Keywords:** cannabis (marijuana), indigenous, remote communities, substance use prevalence, cannabis abstinence, cannabis cessation

## Abstract

**Background:** Cannabis harms among Indigenous populations in Australia, New Zealand, Canada and the United States may be magnified by poorer health and heavy use. However, little direct evidence is available to evaluate cannabis' impacts. In communities in remote northern Queensland (Australia) where cannabis has become endemic, opportunities to support change were investigated.

**Methods:** Opportunistically recruited participants (aged 15–49 years) discussed their cannabis use history in interviews in two waves of population sampling in Cape York (Queensland). Wave 1 included 429 people (235 males and 194 females); and wave 2 included 402 people (228 males and 174 females). Current users (used cannabis during the year before interview) described frequency of use, amount consumed, expenditure and dependence symptoms. Other substance use was recorded for 402 people at wave 2.

**Results:** Wave 1: 69% reported lifetime use and 44% current use. Males (55%) were more likely than females (30%) to be current users (*P* < 0.001). Most (96%) current users described at least weekly use; nearly half (48%) were “heavy” users (≥6 cones/session at least once/week) and 77% met cannabis dependence criteria. Three communities spent up to $AUD14,200/week on cannabis, around $AUD2.0 million/year, or around 9% of community people's total income on cannabis. The majority (79%) of current users wanted to quit or reduce their cannabis use. Wave 2: no difference was observed in the proportion of lifetime (69%, |z| = 0.04, *P* = 0.968) or current cannabis users (39%, |z| = 1.39, *P* = 0.164); nor current use among males (71%, |z| = 0.91, *P* = 0.363) or females (62%, |z| = 0.36, *P* = 0.719). However, a significant reduction in current users by 15% (|z| = 2.36, *P* = 0.018) was observed in one community. Of 105 wave 1 current users re-assessed in 2, 29 (27%) had ceased use. These participants reported cost and family commitments as reasons to change and that social support and employment enabled abstinence. Current and lifetime cannabis use were closely associated with all other substance use, particularly tobacco and alcohol (both *P* > 0.001).

**Conclusions:** High rates of heavy cannabis use in remote Australian Indigenous communities warrant action. Successful cessation among some individuals suggests that significant opportunities are available to support change even where cannabis use may be endemic.

## Introduction

While cannabis remains the most widely used illicit substance worldwide, its use has generally decreased in countries like the United States, Canada, New Zealand and Australia (1). In contrast, rates of cannabis use in the Indigenous populations of these developed economies are 1.3–1.9 times higher than respective national averages (2–5). Systematic evidence is lacking on the specific impacts of cannabis in Indigenous populations, and on how to assist Indigenous peoples to reduce harms.

In the general population, cannabis use is associated with symptoms of: anxiety (6), depression (6–8), dependence (6, 7, 9), and withdrawal (10–12), acute cognitive impairment (13), possible long-term cognitive impairment (14), and schizophrenia (13, 15–17); with evidence that cannabis *causes* psychosis (15, 18–20) becoming stronger (17, 21, 22). Normalization of cannabis use within some sectors of the community (23), and polarized debates about cannabis policy (24), may have diverted attention from its impacts in marginalized and impoverished populations, where harms from most forms of substance use are magnified by the higher prevalence of heavier, riskier patterns of use. Heavier use and significant mental health impacts of cannabis are known in American Indian populations (25), for instance, with very early uptake first nations youth in the United States (26, 27) and Canadian (28). Indigenous Australians, according to national surveys, use cannabis at around 1.6 times the national rate (22). However, these estimates do not include the most remote community populations, such as those in Australia's far north (Northern Territory and north Queensland). These populations are among the more severely disadvantaged and socially excluded populations in the country and have a disproportionate share of a largely preventable chronic disease burden, including that linked with substance misuse (29).

Cannabis became more readily available in remote Indigenous Australian communities just over 20 years ago. Its use was undetected in the 1980s (30) and surged from the late 1990s to as high as 60% in some age groups, more than double national rates (31). Even with such brief exposure, in the Northern Territory's remote Arnhem Land region, high proportions of young users continued to report regular cannabis use between 2001 and 2006 (32, 33). Such use was associated with dependence (33), depression (7), auditory hallucinations, suicidal ideation (7, 32), and imprisonment (34, 35). In similar remote communities in north-eastern Australia (Queensland's Cape York), cannabis users and the communities in which they live may also suffer a heavy burden of cannabis-related harms (36, 37).

Cannabis use is a neglected public health issue in Australia's remote Indigenous communities (38), despite their consistently expressed concerns about its impacts (35, 39). This paper provides evidence from a survey of cannabis use in remote Indigenous communities in north Queensland that describes patterns of use, harms and attitudes toward cannabis.

## Methods

### Hypotheses

Data included in the present analysis comprises semi-structured interviews conducted with participants before and after a community level intervention, with additional participants recruited at the second time-point. It is, therefore, not a before and after study, but represents a sizable sample from each site in two waves of sampling. This study hypothesized:

1. An overall reduction in current users as a result of growing awareness of cannabis harms suggested in the consultations, as well as social marketing activities that occurred between sampling waves.
2. Qualitative examination of those who had ceased using cannabis between the first and second waves would suggest common important factors influencing their decisional balance.

### Setting

Cape York in far north Queensland covers ~211,000 km^2^ with a population of around 20,000 (outside its major regional center and towns). Included are 11,700 Aboriginal and/or Torres Strait Islander (Indigenous) Australians living in 12 very small, self-governing communities with populations ranging from <200 to 2,500 people. Although English is widely spoken, it is usually a second language (or a creole), and many traditional practices are maintained (40). Vehicle access is via unsealed roads, which close for several months in the annual wet season.

Consultation throughout 2007–2008 established community permissions for the study (36). Communities were selected to broadly represent the contemporary settlement pattern for Indigenous people in the region: one near a regional center (Community A); another on Cape York's wet tropical east coast (Community B); and a third on the west coast in drier savannah country (Community C). The three study communities had a combined Indigenous population of 2,187, with 1,172 of these aged 15–49 years at census in 2011 (41).

Queensland Government departments of Health, Education and Police have a presence in all communities. All communities have primary healthcare clinics (PHCs) staffed by allied health workers, including drug and alcohol treatment workers, who periodically fly in from regional centers during the working week. The PHCs also employ local Indigenous health workers and nurses who live in the communities.

There is no published evidence available for the use of illicit drugs other than cannabis in these communities. The sale of alcohol is locally prohibited and its possession and carriage has been tightly restricted since 2008 across Cape York (42).

### Participants and sampling

#### Participant recruitment

**Figure 1:**
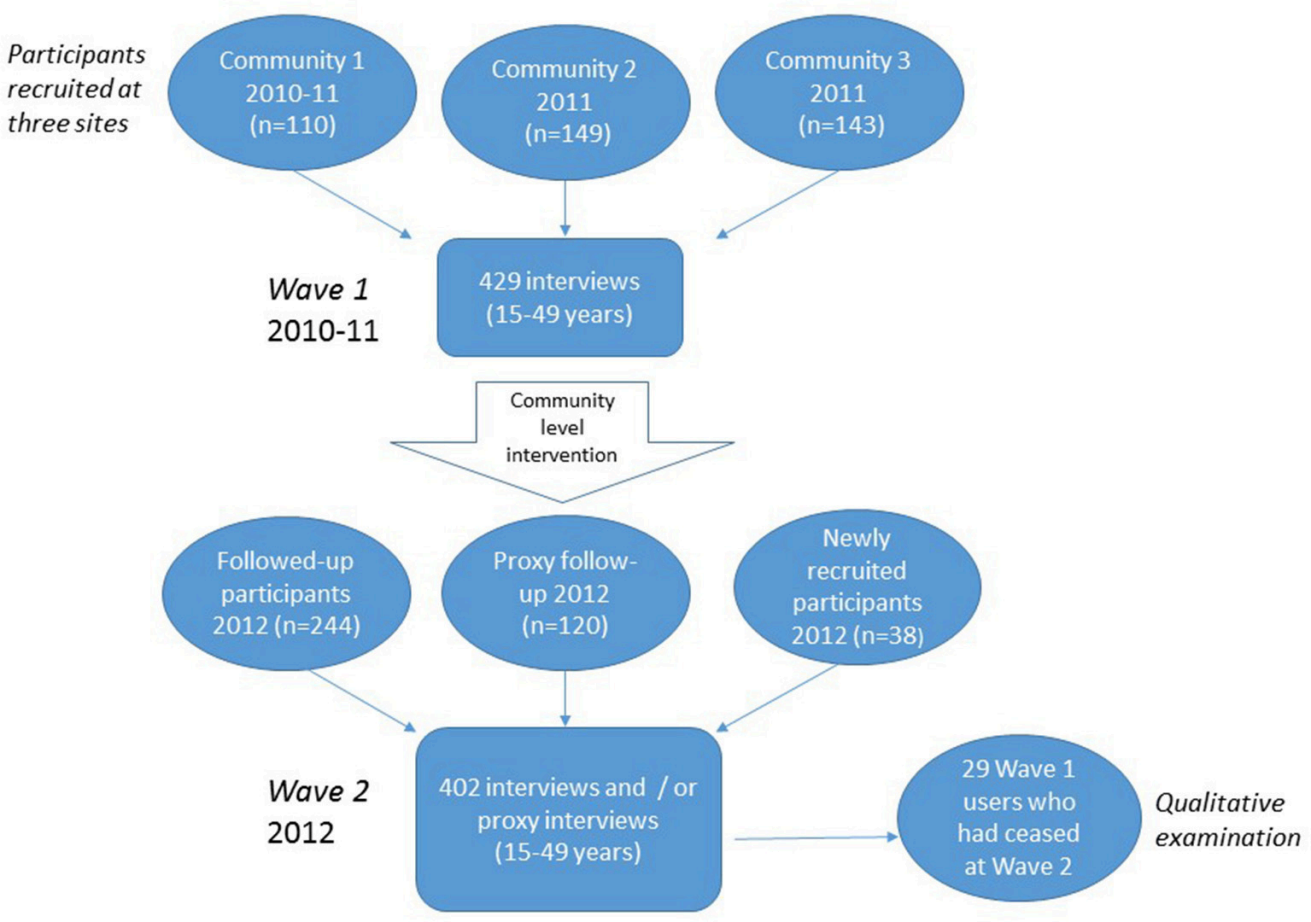
Inclusion of data from three Cape York communities in two waves of sampling 2010–2012.

Data collection at two time-points and its and use in the analysis is shown in Figure 1. Participants were approached for the first wave of interviews between May 2010 and October 2011 as a baseline for a community level demand reduction program. The second wave occurred between May and December 2012, including those participants who were followed up as well as newly recruited participants. This analysis uses all available data from the two waves of sampling to explore inter- and intra-community variations. In practice, because of the brief time between samples in each community, sampling was more-or-less continuous across the three communities as a whole. Research staff visited the communities for 3–5 days, traveling from the regional center (Cairns, 800–1,000 km by road). With stigmatized or illegal behaviors the subject of interest, random sampling in these small community settings is ethically unsustainable (32–34). Recruitment was therefore opportunistic, following strategies used in the Arnhem Land (NT) studies (31, 34), and targeting younger age groups. Researchers alerted communities at least 2 weeks in advance of visits, spending 3 to 5 days in the community each time. Project personnel approached participants outside of the PHC, the community store or in the street and at work places and homes, usually with the paid assistance of a local person.

#### Wave 1 interviews

We used a conversational approach, employed routinely in these localities to work across cultural barriers (33). Semi-structured interviews documented demographics, and lifetime cannabis use. Interviews lasted from 10 min—if participants had little or no experience with cannabis or offered only brief responses— to 30 min, if participants engaged in rich “yarning” about their experiences with cannabis. Current users were asked about frequency of use, age of first/last use, estimated quantity used and weekly expenditure on cannabis. Five severity of dependence scale (SDS) questions were administered to current users (40). Open-ended questions elicited qualitative information from current and former cannabis users about any intentions to change and reasons why.

#### Wave 2 interviews and proxy assessments

In the second wave, researchers recruited new participants to the study and attempted to follow-up all of the original participants. Proxy reports for current or former cannabis use were sought at wave 2 for all participants interviewed at wave 1. Proxy data for use status was used where the participant could not be interviewed at wave 2, a strategy used previously in Arnhem Land (43). In addition to lifetime cannabis use, participants provided data about current and lifetime use of alcohol, tobacco, volatile substances, and other illicit drugs. The SDS questions were administered to current users where the participants consented.

### Data

#### Cannabis use

As in previous studies (32), self-reported cannabis use was described as “never” (never tried cannabis), “former” [tried cannabis once or had not smoked it for ≥12 months, consistent with national data (22)] and “current” (had tried or used cannabis within the past 12 months). Mean duration of use, calculated from age first used and age last used, took account of any significant breaks due to e.g., hospitalization, pregnancy, working or studying away from the community, prison or detention.

#### Frequency of cannabis use–current users

Self-reported frequency of cannabis use among current users was categorized as in previous studies: “daily” (5–7 times/week), “weekly” (1–4 times/week), or “monthly” (1–2 times/month) (33).

#### Defining “heavy” cannabis use

Reported quantities of cannabis used ranged widely from “*one cone a session*” to “*more than twenty*,” making precise quantification difficult. To address a wide diversity of consumption levels and patterns (32), “heavy” use was defined as ≥6 cones per session at least once per week, in line with criteria used in the Arnhem Land (NT) studies.

#### Weekly expenditure on cannabis

Current users were asked to estimate the usual number of “foils” or “sachets” purchased weekly or fortnightly, the price paid ($AUD) and how many people they shared with.

#### Cannabis dependence

Dependence in current users was assessed using the five-item SDS, with scores depicted as a colored chart to address literacy barriers and using a cut-off score ≥3 symptoms experienced in the preceding 3 months (40).

#### Defining “trying to quit”

Further questions, asked of current users who expressed any desire to change, distinguished those who wished to reduce cannabis from those who wished to stop altogether. “Trying to quit” included those who reported current active quit attempts or who reported avoiding cannabis use at some time during the preceding 12 months.

#### Reasons for cessation

Qualitative examination of a subset of interviews with participants who were current users at wave 1 but had ceased cannabis use at wave 2, summarized the principal reasons reported for quitting, barriers to quitting and the resources used to support quitting.

### Data analysis

Quantitative data was analyzed in SPSS (IBM Corp. Released 2017. IBM SPSS Statistics for Windows, Version 25.0. Armonk, NY: IBM Corp). Qualitative data was stored in spreadsheets and imported into NVivo™. Categorical data were compared using the Chi square statistic with 95% confidence intervals. Ordinal variables were compared using the Wilcoxon rank-sum test.

### Ethics approval

The Human Research Ethics Committees of James Cook University and the Cairns and Hinterland Health Services District provided ethical approvals. Study results were provided back to the study communities and their lead agencies after the survey was completed in order to stimulate local action and advocacy among key stakeholder groups.

## Results

### Sample

In total, 429 participants aged 15–49 years were interviewed in the first wave of data collection, equivalent to 37% (= 429/1,172) of the estimated total community populations in this age group. The sample included 55% males (*n* = 235) and 45% females (*n* = 194). This differed from the 2011 census (43) proportions of 49% males and 51% females (|z| = 2.06, *P* = 0.033) in these age groups in the study communities. The proportion of participants (49% = 203/429) aged 15–24 years in the sample was considerably greater than recorded in the census (28%) (|z| = 7.28, *P* < 0.001).

In the second wave, approximately 12 months later, data were collected for 402 people, including: 244 wave 1 participants who completed follow-up interviews; 120 proxy assessments of wave 1 participants; and 38 new participants. Proportions of males (*n* = 228, 57%) and females (*n* = 174, 43%) were similar to the first wave (|z| = 0.56, *P* = 0.575), and similarly different to the 2011 census (|z| = 2.68, *P* = 0.007). Overrepresentation of younger participants aged 15–24 years (37% = 149/402) compared to the census data (28%) (|z| = 3.42, *P* > 0.001), was more pronounced than at wave 1 (|z| = 2.99, *P* = 0.003).

### Patterns of use at wave 1

#### Reported lifetime cannabis use varied across the communities and gender groups

The proportions of the sample reporting cannabis use at least once in their lifetime ranged from 54 to 81% across the communities (69% overall) (Table 1A). In community C, almost half the participants (46%) had never used cannabis, whereas in community A, this was true of fewer than one in five (19%) (Table 1A).

**Table 1A:** Demographic characteristics and cannabis use in 429 people (235 males and 194 females, aged 15–49 years) interviewed at wave 1 in three Cape York communities, far north Queensland, Australia, 2010–2011.

		**Community A *n* = 139**	**Community B *n* = 135**	**Community C *n* = 155**	**Total *n* = 429**	P^*^
Gender	Female	63 (45.3%)	65 (48.1%)	66 (42.6%)	194 (45.2%)	
	Male	76 (54.7%)	70 (51.9%)	89 (57.4%)	235 (54.8%)	*P* = 0.636
Age group	15–24	65 (46.8%)	66 (48.9%)	72 (46.5%)	203 (47.3%)	
	25–34	39 (28.1%)	42 (31.1%)	39 (25.2%)	120 (28.0%)	*P* = 0.543
	35–49	35 (25.2%)	27 (20.0%)	44 (28.4%)	106 (24.7%)	
Cannabis use	Non–user	27 (19.4%)	36 (26.7%)	72 (46.5%)	135 (31.5%)	
	Former user	38 (27.3%)	40 (29.6%)	28 (18.1%)	106 (24.7%)	*P* = 0.001
	Current user	74 (53.2%)	59 (43.7%)	55 (35.5%)	188 (43.8%)	

* Pearson chi^2^

**Table 1B:** Demographic characteristics and cannabis use in 402 people (228 males and 174 females, aged 15–49 years) with data at wave 2 (followed-up, 244; proxy, 120; or newly recruited, 38) in three Cape York communities, far north Queensland, Australia, 2012.

		**Community A *n* = 110**	**Community B *n* = 149**	**Community C *n* = 143**	**Total *n* = 402**	P^*^
Gender	Female	50 (45.4%)	65 (43.6%)	59 (41.3%)	174 (43.3%)	
	Male	60 (54.6%)	84 (56.4%)	84 (58.7%)	228 (56.7%)	*P* = 0.796
Age group	15–24	42 (38.2%)	58 (38.9%)	49 (34.3%)	149 (37.1%)	
	25–34	37 (33.6%)	57 (38.3%)	47 (32.9%)	141 (35.1%)	
	35–49	31 (28.2%)	34 (22.8%)	47 (32.9%)	112 (27.9%)	*P* = 0.451
Cannabis use (402)	Non-user	25 (22.7%)	30 (20.1%)	71 (49.6%)	126 (31.3%)	
	Former user	43 (39.1%)	53 (35.6%)	23 (16.1%)	119 (29.6%)	
	Current user	42 (38.2%)	66 (44.3%)	49 (34.3%)	157 (39.1%)	*P* = 0.001

* Pearson chi^2^

Overall, males (77%) were more likely to report lifetime use than females (59%) (*P* < 0.001). Age-standardized rates were 78% of males, 52% of females and 65% overall. However, the differences in the crude proportions of lifetime users also varied across communities: A (86% males, 75% females, *P* = 0.105); B (79% males, 69% females, *P* = 0.299) and with women less likely to have ever tried cannabis in community C only (69% males, 33% females, *P* < 0.001).

The proportion of lifetime users (71%) in the younger participants (aged 15–24 years) was similar (66%) to older participants (aged 25–49 years) (*P* = 0.221) and varied little across the communities.

#### Current users

Males were generally more likely to report current cannabis use in the sample in all three communities: A (66% males, 38% females, *P* = 0.001); B (51% males, 35% females, *P* = 0.060) and C (49% males, 17% females, *P* < 0.001) and around three times more likely overall (55% males, 30% females, *P* < 0.001). Age standardized rates were 55% for males and 26% for females, 40% overall.

#### Age of uptake and duration of use among current users

Table 2A describes the patterns of cannabis use among 188 current users, comprised of 69% males (*n* = 130) and 31% females (*n* = 58). Their median age was 24 years, with males around 2 years older than females (*P* = 0.063). Participants had used cannabis for up to 30 years (median = 11 years for males, = 6 years for females, *P* = 0.003). Age of first use was similar in males and females (median = 16 years, *P* = 0.714) (Table 2A).

**Table 2A:** Patterns of cannabis use by gender in 188 current users (aged 15–49 years) interviewed at wave 1 in three Cape York communities, far north Queensland, Australia, 2010–2011.

		**Male *n* = 130**	**Female *n* = 58**	**Total *n* = 188**	P^*^
Median age	Years (min–max)	25 (16–49)	23 (15–47)	24 (15–49)	|z| = −1.86, *P* = 0.063^†^
Median age of first use	Years (min-max)	16 (8–30)	16 (12–37)	16 (8–37)	|z| = −0.37, *P* = 0.714
Median duration of use	Years (min-max)	10.8 (1.20–28.9)	6.20 (0.20–30.0)	7.95 (0.20–30.0)	|z| = −3.50, *P* = 0.003
Heavy user	≥6 cones/session at least once/week	56 (47.1%)	25 (51.0%)	81 (48.2%)	*P* = 0.640
Frequency	Daily	71 (55.0%)	25 (45.5%)	96 (52.2%)	
	Weekly	54 (41.9%)	26 (47.3%)	80 (43.5%)	*P* = 0.289
	Monthly	4 (3.10%)	4 (7.27%)	8 (4.35%)	
Median weekly spending	$AUD/week (min-max)	50 (0–1050)	31 (0.0–350)	50 (0.0–1050)	|z| = −2.45, *P* = 0.014^†^
Severity of Dependence Scale	≥3 symptoms	95 (76.6%)	35 (66.0%)	130 (73.4%)	*P* = 0.145
Intentions toward cannabis	None	65 (56.0%)	29 (60.4%)	94 (57.3%)	
	Trying or wishes to quit	51 (44.0%)	19 (39.6%)	70 (42.7%)	*P* = 0.606

* *Pearson chi^2^ unless otherwise specified*.

† *Wilcoxon rank-sum test*.

#### Patterns of current use

Almost half (48%) of the 168 current users, for whom information was available were “heavy users” with similar proportions in males (47%) and females (51%) (*P* = 0.640 Table 2A). Half (52%) of 184 current users reported using cannabis on a daily basis, another 43% used it on a weekly basis. The majority used cannabis regularly, with little difference between males (97%) and females (93%) (Table 2A).

#### Style of cannabis use and expenditure by current users

The nominated preferred style of use in all three communities was hand-made “bucket bong,” a negative pressure device constructed from a bottomless bottle with a cone piece inserted into the lid, plunged into a larger container of water to draw the smoke in to be inhaled from the bottle. Almost all current and former users reported that they mixed tobacco with cannabis.

Across the communities, participants reported that cannabis was purchased from dealers (i.e., not cultivated in the community), with further distribution within the community through on-selling or sharing. Cannabis was mostly supplied in aluminum “foils” or plastic “sachets” with prices ranging from $AUD20 to $AUD50 per unit. Users reported considerable variation in the unit quantity and quality of cannabis material, often premixed with tobacco.

Males tended to spend more on purchasing cannabis than females, $AUD50/week compared with $AUD31/week (|z| = 2.45, *P* = 0.014, Table 2A). With current users comprising 44% (= 188/429) of the sample, this means there may be 514 (= 188/429^*^1,172) current users in the 15–49 years age group in the three communities overall. A crude estimate of total expenditure on cannabis in this age group in these three communities is $AUD39,000 per week (= 514/188^*^$AUD14,200/week) equivalent to just over $AUD2.0 million/year.

#### Severity of cannabis dependence (SDS) in current users

Three quarters (73%) of the current users met criteria for cannabis dependence (SDS ≥ 3), with similar proportions in males (77%) and females (66%) (*P* = 0.145), Table 2A). Data not shown indicates that similar proportions of current users met dependence criteria in both the younger (74%, 15–24 years) and older (73%, 25–49 years) age groups (*P* = 0.813). Those in the “heavy use” category were no more likely than other current users to meet criteria for cannabis dependence (*P* = 0.787). Current users who met dependence criteria, however, spent more on cannabis (median spend = A$50/week) than those who did not (median spend = A$38/week) (|z| = 2.09, *P* = 0.036).

### Patterns of use at wave 2

#### Reported lifetime cannabis use varied across the communities and gender groups

Shown in Table 1B, the proportions of the sample reporting cannabis use at least once in their lifetime ranged from 79 to 50% across the communities (69% overall) which was not different to wave 1 (|z| = 0.04, *P* = 0.968). In community C, half (50%) had never used cannabis, whereas in community A, this was true of less than a quarter (23%). No significant differences in proportions of non-users were recorded between wave 1 and wave 2 samples across the communities: A (19% wave 1, 23% wave 2 |z| = 0.64, *P* = 0.522); B (27% wave 1, 20% wave 2, |z| = 1.30, *P* = 0.194) and community C (46% wave 1, 50% wave 2, |z| = 0.55 *P* = 0.582).

Overall, at wave 2, males (78%) were more likely to report lifetime use than females (56%) (*P* < 0.001). Age standardized rates of lifetime cannabis use were 80% for males and 53% for females overall. However, the differences in the crude proportions of lifetime users also varied across communities: A (85% males, 68% females, *P* = 0.034); B (86% males, 72% females, *P* = 0.043) and strongest in community C (67% males, 27% females, *P* < 0.001). As for the sample at wave 1, the proportion of lifetime users at wave 2 (68%) in the younger participants (aged 15–24 years) was similar (69%) to older participants (aged 25–49 years) (*P* = 0.947) and varied little across the communities.

#### Current users at wave 2

No statistically significant difference was detected in the proportion of cannabis users in the overall sample at wave 1 (*n* = 188, 44%) compared to wave 2 (*n* = 157, 39%) (|z| = 1.39, *P* = 0.164). Shown in Table 2B, age standardized rate of current use among males was 52% and 21% for females. A significant reduction of 15% in current users (53 to 38%, |z| = 2.36, *P* = 0.018) was recorded in community A. The proportion of heavy users in the sample at wave 2 (63%) was higher than at wave 1 (51%) (|z| = 2.40, *P* = 0.016). Compared to wave 1, the proportion of males (71%, |z| = 0.91, *P* = 0.363), females (62%, |z| = 0.36, *P* = 0.719) and younger users (69%, |z| = 0.62, *P* = 0.535) reporting more than three symptoms of dependence were not different at wave 2. The median weekly spending at wave 2 of $50 per week was not different to wave 1 overall, with a similar difference between males and females ($55 and $30 per week, respectively (|z| = 2.57, *P* = 0.010).

**Table 2B:** Patterns of cannabis use by gender in 157 current users (aged 15–49 years) with wave 2 data, either followed up (88) or newly recruited (27) in three Cape York communities, far north Queensland, Australia, 2010–2011.

		**Male *n* = 130**	**Female *n* = 58**	**Total *n* = 188**	P^*^
Median age	Years (min–max)	27 (16–49)	25 (15–46)	26 (15–49)	|z| = −2.06, *P* = 0.039^†^
Median age of first use	Years (min-max)	16 (8–30)	16 (12–37)	16 (8–37)	|z| = −0.51, *P* = 0.607
Median duration of use	Years (min-max)	8.1 (1.20–28.9)	7.15 (0.20–26.1)	7.80 (0.20–28.9)	|z| = −1.80, *P* = 0.072
Heavy user	≥6 cones/session at least once/week	50 (63.3%)	18 (62.1%)	68 (63.0%)	*P* = 0.907
Frequency	Daily	35 (43.8%)	9 (32.1%)	44 (40.7%)	
	Weekly	41 (51.2%)	16 (57.1%)	57 (52.8%)	*P* = 0.393
	Monthly	4 (5.00%)	3 (10.7%)	7 (6.48%)	
Median weekly spending	$AUD/week (min–max)	55 (0–800)	30 (0–300)	50 (0–800)	|z| = −2.57, *P* = 0.010^†^
Severity of dependence scale	≥3 symptoms	56 (70.9%)	18 (62.1%)	75 (63.0%)	*P* = 0.968
Intentions toward cannabis	None	31 (56.4%)	11 (47.8%)	42 (53.8%)	
	Trying or wishes to quit	24 (43.6%)	12 (52.2%)	36 (46.2%)	*P* = 0.619

* *Pearson chi^2^ unless otherwise specified*.

† *Wilcoxon rank-sum test*.

*No data for proxy (n = 42)*.

#### Lifetime and current substance use at wave 2

Lifetime use of cannabis was linked with lifetime use of tobacco, alcohol and other illicit substances (*P* < 0.001). Current use of cannabis (39%) was strongly associated with current use of tobacco (74%, *P* < 0.001) and alcohol (64%, *P* < 0.001). Seven participants reported current inhalant use and all of these were current cannabis users.

### Qualitative information

#### Quit intentions among current users

Of 188 current users at wave 1, 164 provided information about their intentions to stop or reduce cannabis use. Overall, 70 current users (43%) indicated they were trying or wanted to quit (Table 2A), including 10% (*n* = 16) actively trying to quit at wave 1. At wave 2, 46% (36/78) said they wanted to change.

#### Reasons for change among participants who ceased using cannabis between wave 1 and wave 2

Twenty-nine participants who were “current users” at wave 1 (2011) were no longer using cannabis at wave 2 (2012). This included 14 women and 15 men, with no obvious differences in distribution across age groups, genders or communities.

Among 15 men, 11 said they wanted to quit, including five who were then making a quit attempt when first interviewed in 2011. Two had said they wished to cut down and only one had said that he did not want to quit. Nine of these men explained their reasoning: it was too expensive or a waste of money (3); family as the principal reason for quitting, particularly concern for their children (4); and health reasons or getting older (2). Among 14 women who had ceased cannabis use, 5 had indicated a desire to quit at wave 1, including 2 actively trying to quit. A further two said they would like to cut down and three who did not answer the question nonetheless discussed earlier quit attempts. Seven explained their reasoning: family (including children and pregnancy) (4) or for work (3).

Only men in this group of successful quitters mentioned the expense of cannabis as a reason to stop, perhaps reflecting the tendency for men to spend more on cannabis and suggesting that women are probably more likely to source cannabis from partners or family members. One young man described how he demonstrated for himself how much money he was wasting by collecting the packaging:

“*Started collecting sachets this year. Ten sachets is $500. I've spent $1000 on that silly thing this year.”*

Resources that enabled cessation mentioned by these 29 participants included: keeping busy with work; childcare or cultural activities; or spending time with non-using friends and family. For example, a young woman said that she would “*get help from sisters and brothers because they understand”* (Table 3). Conversely, cue exposure and normalization was a barrier to cessation for the young man referring to “*other boys, temptation*” (Table 3 “Barriers in context”).Only one person mentioned health services as a possible strategy to support cessation.

**Table 3:** Participant reasoning for successful quit attempts between wave 1 and wave 2 and enablers and barriers mentioned.

	**Reasoning for quitting**	**Enabling context**	**Barriers in the context**
Women	*Had a baby and needed to go my own way*. *I want to give up and focus on work.* *…because looking after lots of kids*.	*Thinking about giving up, would like to get help from sisters and brother because they understand.* *Job would keep me from staying in the house smoking*	[When cannabis unavailable] *makes you feel like you want to go look for more [cannabis]. Stressing out*.
Men	*Spending money on wrong things - no food in the house*. *It's all about cash, that thing getting expensive* *Daughter told me to stop smoking, she was 3 at the time.* *Used to smoke all day long. I've given up for my son.* *Realised important things in life were work and family*	*I never buy it;* *Long as I got the job I've got no stress - always up early*. *Mum wants me to give up*. [I want to] *slowly give up–work keeps you occupied*. *Get people busy—mentor younger boys and men*.	*Fighting and stressing out when* [there is] *no gunja, look for credit if none get wild with the dealer* *Relaxes me… want to get stress down before I bring it out on my family* *Pulled out from school for fighting at age 14 and became a steady smoker since*. *Other boys temptation Calms you and you're not annoyed*.

This 22-year old man described a variety of arguments and opportunities that he believed would support cannabis cessation:

“*Put food on the table; buy power card; get the outstations going; get cattle; hunting. [It causes] fighting and stressing out…”*

Selected quotes summarized in Table 3 describe reasons for quitting, and similar enablers and barriers in context.

## Discussion

Although there was some variation in the crude rates across the participating communities in Cape York, age-standardized rates of lifetime cannabis use of 65% (78% for males and 52% for females) found in this study are higher than in the general Australian population where just under half of those in comparable age groups report lifetime use (22). The age-standardized proportions of current cannabis users at wave 1 in the study (55% of males and 26% of females aged 15–49 years), 40% overall, are similar to 67% males and 22% females (aged 13–36 years), and around 50% overall, documented in Arnhem Land (NT) in 2001 (34), most of whom were still users at follow-up in 2005–06 (38). Again similar to Arnhem Land (34), around half (48%) of the current users in this study were categorized as “heavy users,” with most (>90%) using cannabis at least weekly (Table 2A).

The highest rate of past year cannabis use reported nationally in 2010 was 25% for males (aged 20–29 years) and 19% for females (aged 18–19 years) (2). In this study, however, almost all the current cannabis users reported at least past month use, compared with < 6% (aged ≥14 years) past month use nationally. It is noteworthy that between 1998 and 2007, in Australia generally, there was a sharp decline in cannabis use from 17.9 to 9.1%. This included a decline from 36.5 to 13.8% in the NT and from 17.5 to 9.5% in Queensland (2). At the same time in the NT (31), however, and now documented in far north Queensland, cannabis use probably increased to become the significant challenge for cannabis users and the general community population that it is today.

### Opportunities to address cannabis use

Although crude rates of lifetime use found in the study were very high (69%), it is encouraging that more than a third of lifetime users had succeeded in quitting in the samples. Among 29 people who had ceased using cannabis at wave 2, pregnancy among women (44) and perceived barriers of withdrawal stress (45), limited recourse to clinical support and the importance of the social context (46) have been reported as challenges to quitting cannabis in other populations. Adults in other populations have seldom reported employment and financial impacts cessation drivers, perhaps reflecting the extremely limited employment opportunities and young age of many of the users in the current study.

Self-selection of a supportive environment, important in self-initiated cessation (44), is difficult in remote Indigenous Australian communities. Cannabis use is normalized among close-knit family groups living in generally overcrowded housing. Cue exposure is high and opportunities for meaningful long-term employment are limited. Intensified cessation support from health services is warranted but, as noted in other high-risk populations (46, 47), these must be proactive in incorporating latent and active strategies already embedded in the local social context (13). For example, interventions may incorporate the effects of widespread trauma (48), cultural perspectives (49), and social support (50). Work readiness programs may assist those seeking to quit, especially if aligned with genuine employment opportunities.

Widespread community concern about youth uptake and its effects on mental health is a prevention opportunity. It is generally recognized that young people should be advised that early cannabis use may bring serious long-term harms (51) and, as the following quote demonstrates, Indigenous community members recognize this, exemplified by the following quote from an interview with a woman in her early twenties:

“*Young kids start and then build up and don't stop. Get addicted early.”*

Efforts to reduce adolescent uptake also need to target the social context in which cannabis is used to add strength to a focus on individual decisional balance (52). Social marketing to support others' cessation might be used to better effect than raising awareness of individual level harms. Resourcing and policy to support youth engagement in school or training and strong social supports are critical.

Local financial impacts are significant, with high cost a frequently reported negative consequence for current users (36, 53). The crude estimate of the local cannabis trade at ~$AUD39,000/week in this small population of around 2,187 people is similar to estimates a decade ago for NT communities ($AUD19,000–$AUD32,000/week for 2,649 people) (33). The gross annual income of the Indigenous members of the three communities in this study is approximately $25.1 million (43), of which, the local cannabis market may constitute around 9%. This parallels the widely voiced concern about broader adverse impacts on families and community and concerns about financial impacts reported during earlier consultation (35). Since similar impacts have been documented for similar remote NT communities (33) this information could be incorporated into motivational strategies and general social marketing to encourage support for those seeking to quit.

### Cannabis dependence, “heavy use” and weekly expenditure

The association between cannabis dependence [probably reinforced by nicotine (54)] and weekly expenditure on cannabis by current users (*P* = 0.023) confirms concerns about adverse financial impacts of trafficking and addiction in users. The expected association between cannabis dependence and “heavy use” was not apparent, perhaps because of the narrow range of levels of use found. Notwithstanding the challenges of measuring “heavy use,” the precise nature of the experience of cannabis dependence in these settings, where resources such as cannabis and money to purchase it are shared, requires further research into the social underpinnings of addiction in this population.

### Study limitations

This sample was not randomly selected, but included more than one-third of males and females aged 15–49 years in the study communities, and nearly half of the 15–24 year-olds at each site. While there was bias in the overall sample toward younger and hard-to-reach males, the proportions of males and females in these age groups were broadly consistent across the communities (Tables 1A,B). Therefore, gross differences between communities in prevalence of cannabis use are less likely to be distorted by this sampling bias although the overall sample results may not be generalizable.

## Conclusions

The high rate of heavy and problematic cannabis use in remote Indigenous Australian communities is clearly not isolated to one part of northern Australia as reported in the limited available literature on the topic (32, 33). Substantial numbers of users in our sample were seeking to quit, which may be encouraging for people living in the participant communities. We can no longer overlook the opportunities revealed in this modest study to assist similar community populations to reduce cannabis use and address its local harms.

Indigenous populations living in similarly isolated communities elsewhere in the world, where fundamental asymmetries of social and economic power are most stark, may be especially vulnerable to experiencing cannabis harms (29, 37). Indigenous vulnerability to heavy episodic substance use reflects socioeconomic disadvantage relative to the wider economic situation (55) in a population along with a range of social (56), family (20, 39), and systemic factors (57–59). The influences of these factors on regional and ethnic variations in rates of cannabis use within nations like Australia are poorly understood and should be further investigated (21).

## Author contributions

VG was the lead project officer during wave 1 of data collection and the final part of wave 2. She performed the statistical calculations and wrote the first draft. AC designed the study, advised on statistical analysis methods, and approved the final manuscript.

### Conflict of interest statement

The authors declare that the research was conducted in the absence of any commercial or financial relationships that could be construed as a potential conflict of interest.
